# Identification and Expression of *SAUR* Genes in the CAM Plant *Agave*

**DOI:** 10.3390/genes10070555

**Published:** 2019-07-23

**Authors:** Gang Deng, Xing Huang, Li Xie, Shibei Tan, Thomas Gbokie, Yaning Bao, Zhouli Xie, Kexian Yi

**Affiliations:** 1 School of Agriculture, Yunnan University, Kunming 650504, China; 2 Environment and Plant Protection Institute, Chinese Academy of Tropical Agricultural Sciences, Haikou 571101, China; 3 College of Forestry, Hainan University, Haikou 570228, China; 4 College of Plant Protection, Nanjing Agricultural University, Nanjing 210095, China; 5 College of Plant Science and Technology, Huazhong Agricultural University, Wuhan 430070, China; 6 Department of Genetics, Development and Cell Biology, Iowa State University, Ames, IA 50011, USA

**Keywords:** *Agave*, *SAUR*, phylogeny, gene expression, abiotic stress, biotic stress

## Abstract

*Agave* species are important crassulacean acid metabolism (CAM) plants and widely cultivated in tropical areas for producing tequila spirit and fiber. The hybrid H11648 of *Agave* ((*A. amaniensis* × *A. angustifolia*) × A. *amaniensis*) is the main cultivar for fiber production in Brazil, China, and African countries. *Small Auxin Up-regulated RNA* (*SAUR*) genes have broad effect on auxin signaling-regulated plant growth and development, while only few *SAUR* genes have been reported in *Agave* species. In this study, we identified 43, 60, 24, and 21 *SAUR* genes with full-length coding regions in *A. deserti*, *A. tequilana*, *A*. H11648, and *A. americana*, respectively. Although phylogenetic analysis revealed that rice contained a species-specific expansion pattern of *SAUR* gene, no similar phenomena were observed in *Agave* species. The in silico expression indicated that *SAUR* genes had a distinct expression pattern in *A*. H11648 compared with other *Agave* species; and four *SAUR* genes were differentially expressed during CAM diel cycle in *A. americana*. Additionally, an expression analysis was conducted to estimate *SAUR* gene expression during different leaf developmental stages, abiotic and biotic stresses in *A*. H11648. Together, we first characterized the *SAUR* genes of *Agave* based on previously published transcriptome datasets and emphasized the potential functions of *SAUR* genes in *Agave’s* leaf development and stress responses. The identification of which further expands our understanding on auxin signaling-regulated plant growth and development in *Agave* species.

## 1. Introduction

*Small Auxin Up-regulated RNA* (*SAUR*) family is one of the important gene families that are involved in auxin signaling-regulated plant growth and development [1]. Genes in this family have been reported as a marker gene in soybean, *Arabidopsis*, and tobacco during early auxin responses [2,3,4]. Nowadays, the auxin signaling-related function of *SAUR* genes has also been reported in several other species, including tomato, mung, apple, radish, maize, pepper, rice, cotton, litchi, potato, peach, citrus, ramie, and sorghum [5]. A series of molecular studies in *Arabidopsis* indicate that these genes participate in plant developmental processes, including in cell elongation [6], cell expansion [7,8,9], light signaling [10,11], branch angle formation [12], pollen tube growth [13] and interactions with brassinosteroid [14], gibberellin [15], and ethylene [16]. In other species, the *SAUR* genes are associated with fruitlet abscission in citrus [17], auxin-dependent hypocotyl elongation in tomato [18], auxin synthesis, and transport in rice [19], and starch accumulation in cassava [20] as well. Recently, the rapid development of next-generation sequencing (NGS) allows researchers to obtain and explore more information [21]. For example, genome-wide identification of *SAUR* genes has been performed in rice [22], *Arabidopsis*, maize, sorghum [23], tomato, potato [24], citrus [17], moso bamboo [25], watermelon [26], cotton [27] and poplar [28]. Moreover, most of those contain the species-specific expansion pattern, which probably contributes to the evolution of special traits among different species [23,26]. Consider *SAUR* genes are crucial effectors of hormonal and environmental signals, functional characterization of *SAUR* genes will broaden our understanding in plant growth and development [29,30].

Up till now, only few *SAUR* genes are reported in *Agave* species, despite these species are largely applied in alcoholic beverages, fiber, and food production [31]. It reasons that the genomes of *Agave* are too large to sequence, while the most recent publications on *Agave* transcriptomes provides a great opportunity for their genetic researches [32]. Furthermore, NGS tools are utilized in *Agave* species for further functional gene mining, such as stress-related genes in *Agave deserti*, fructan-related genes in *A. tequilana* [33], fiber-related genes in *Agave* hybrid H11648 ((*A. amaniensis* × *A. angustifolia*) × *A. amaniensis*) [31] and CAM photosynthesis-related genes in *A. americana* [34]. These transcriptome datasets make the identification of *SAUR* genes and evaluation of their phylogenetic relations in *Agave* species to be available. In this study, we select the main cultivar in China, *A*. H11648 to perform further gene expression analysis of *SAUR* genes at different leaf developmental stages and under abiotic/biotic stresses. Therefore, our findings enhance the understanding of the *SAUR* genes on auxin signaling-regulated plant growth, development and stress responses in *Agave* species.

## 2. Materials and Methods

### 2.1. Sequence Retrieval and Subcellular Localization

Fifty-six rice *SAUR* genes were downloaded from public databases [22] and employed as queries to search against *Agave* transcriptomes by TBlastx method [35]. The transcriptomes of *A. deserti*, *A. tequilana*, *A*. H11648 and *A. americana* were selected for sequence retrieval [31,33,34,36]. Target sequences from the four Agave transcriptomes were analyzed for coding sequence with ORF-FINDER [37]. *SAUR* genes of *Agave* with full coding sequences were used for subcellular localization prediction using CELLO software [38].

### 2.2. Phylogenetic Analysis

The proteins of *SAUR* in *Arabidopsis*, rice and the four *Agave* species were utilized for phylogenetic analysis. A maximum likelihood (ML) tree was constructed using MEGA 5.0 software [39]. Bootstrap values were tested for 1000 trails to construct the most parsimonious tree. DNAMAN 7 software was used to predict the conserved domains of *SAUR* [40].

### 2.3. Plant Materials and RNA Extraction

The plants of *A*. H11648 were grown in pots at Environment and Plant Protection Institute, Chinese Academy of Tropical Agricultural Sciences (19.99° N, 110.33° E). Shoot, unexpanded leaf and, expanded leaf were separately collected from 2-year-old plants at different developmental stages. Abiotic and biotic stress treatments were conducted using 1-year-old plants. It has been reported that *A*. H11648 has a high tolerance to heavy metal stress, such as copper and lead [41,42]. Thus, CuSO_4_ and Pb(NO_3_)_2_ solutions were utilized as abiotic stresses for watering plants at the concentrations of 1 g/Kg and 1.3 g/Kg (heavy metal salt/soil), respectively [41,42]. About 2 weeks later, the leaves of plants with treatment were starting curling and collected as samples. Moreover, Zebra disease is the most serious problem of sisal production in China and the pathogen has been identified as *Phytophthora nicotianae* Breda [36,43]. A *Phytophthora nicotianae* Breda strain was inoculated on *A*. H11648 leaves as biotic stress, and the leaves were sampled after 5 days as previously reported [43]. Untreated leaves were also sampled as control. Each treatment was repeated in three individual plants as biological replicates. The collected leaves were immediately placed into liquid nitrogen. A Tiangen RNA prep Pure Plant Kit (Tiangen Biomart, Beijing, China) was used for RNA extraction according to the manufacturer’s protocol. Total RNAs were stored at −80 °C.

### 2.4. Expression Analysis

*SAUR* genes in the four *Agave* species were selected for in silico expression analysis and Reads Per Kilobase per Million mapped reads (RPKM) values in leaves were obtained from previous studies [31,33,34,36]. For qRT-PCR analysis, total RNA of *A*. H11648 were reverse transcribed with GoScript Reverse Transcription System (Promega, Madison, WI, USA). Each qRT-PCR reaction with a final volume of 20 μL contained 0.5 μL gene-specific primers (10 μM), 1 μL cDNA template, 10 μL TransStart Tip Green qPCR Supermix (Transgen Biotech, Beijing, China), 0.4 μL Passive Reference Dye (50×) (Transgen Biotech, Beijing, China) and 7.6 μL ddH_2_O. qRT-PCR reaction was carried out in a QuantStudio 6 Flex Real-Time PCR System (Thermo Fisher Scientific, Waltham, MA, USA) with thermal cycles as follows: 94 °C, 30 s; 94 °C, 5 s and 60 °C, 30 s for 40 cycles; dissociation stage. Each sample was repeated three times as technical repeat. Specific primers for eight *SAUR* genes of *A*. H11648 were designed with Primer 3, together with the *protein phosphatase 2A* (*PP2A*) gene as endogenous control according to a previous study (Table S1) [31,44]. The ΔΔCt method was used for calculating relative expression levels [45].

## 3. Results

### 3.1. Identification and Subcellular Localization of Agave *SAUR* Genes

After sequence retrieval, we found 43, 60, 24 and 21 *SAUR* genes with full-length coding regions in *A. deserti*, *A. tequilana*, *A*. H11648 and *A. americana*, respectively (Table S2). These genes ranged from 234–537 base pairs in the coding region with predicted proteins of 77–178 amino acids. About 148 genes were further analyzed for their subcellular localization (Table S2). As a result, most *Agave SAUR* genes were located in the nucleus or mitochondria (Table 1). And more genes in *A. deserti* and *A. tequilanas* were located in the nucleus or mitochondria than those in *A*. H11648 and *A. americana*. Only a few genes were located in cytoplasm, chloroplast or plasma membrane. The similar numbers of *Agave* genes located in chloroplast and plasma membranes, while more genes in *A. deserti* were located in the cytoplasm than other *Agave* species. Interestingly, two *SAUR* genes were located extracellularly in *A. tequilana* (Table 1).

### 3.2. Phylogenetic Analysis of Agave *SAUR* Genes

**Figure 1:**
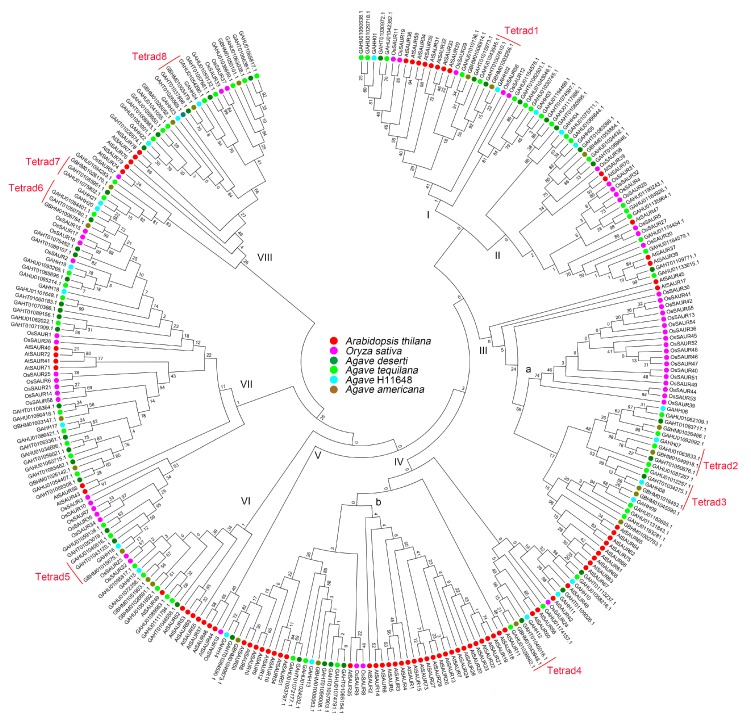
Phylogenetic tree of *SAUR* proteins from *Arabidopsis* (red), rice (pink), *A. deserti* (dark green), *A. tequilana* (green), *A*. H11648 (blue) and *A. americana* (brown). *Agave* homolog tetrads were highlighted in red. The species-specific expansion of *SAUR* genes was highlighted in rice (a) and *Arabidopsis* (b), respectively.

All SAUR proteins in *Arabidopsis* (79), rice (56) and *Agave* species (148) were utilized in the phylogenetic analysis, by which these genes were clustered into eight groups (Figure 1). Typically, *Agave* sequences were grouped together, and eight subbranches (tetrads) contained sequences from the four *Agave* species. *A*. H11648 and *A. americana* shared similar numbers of *SAUR* genes in all groups, while the number of which were much smaller than those in *Arabidopsis*, rice, *A. deserti*, and *A. tequilana* (Table 2). Furthermore, more *Agave* sequences exist in group I, II, and VIII compared with more rice sequences were found in group III and VII and more *Arabidopsis* sequences were observed in group IV and V. Interestingly, 17 rice sequences and 21 *Arabidopsis* sequences were clustered together in group III and IV, which also formed a larger amount than in *Agave* species (Figure 1). About 14 highly conserved amino acid residues of SAUR protein in *Agave* species were identified based on the alignment (Figure S1).

### 3.3. In Silico Expression of *SAUR* Genes in Agave

**Figure 2:**
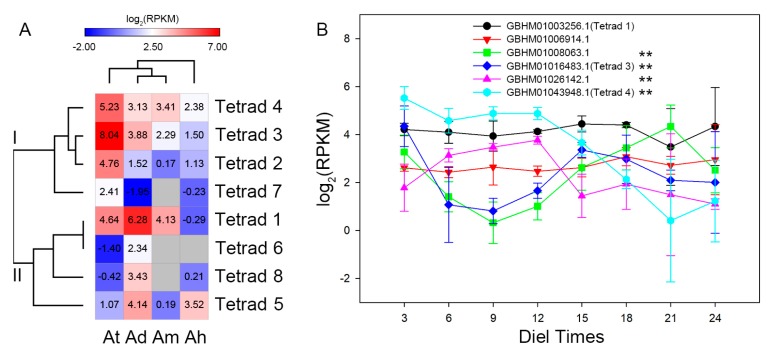
(**A**) The in silico expression of *SAUR* tetrad homologs in the leaves of *A. deserti* (Ad), *A. tequilana* (At), *A.* H11648 (Ah) and *A. americana* (Am) according to previous studies [31,33,34]. Blanked squares represent no expression data. (**B**) The in silico expression of *SAUR* genes across the diel cycle of CAM photosynthesis in *A. americana* according to a previous study [34]. The numbers of *x*-axis represent diel times 3, 6, 9, 12, 15, 18, 21 and 24 h from the beginning of the light period. Differentially expressed *SAUR* genes were highlighted with **. Error bars represent standard deviations.

Based on transcriptomic data, the in silico expression dynamics of *SAUR* genes in *Agave* leaves were obtained (Table S2). We further compared the expression patterns of *SAUR* genes in the eight tetrads, from which two expression modes were characterized (Figure 2A). In mode I, four *SAUR* genes of *A. tequilana* showed higher expression levels than other three species, while four *SAUR* genes of *A. deserti* were highly expressed than others in mode II. Remarkably, *SAUR* genes in *A*. H11648 showed a more distinct expression pattern than other species. Furthermore, four *SAUR* genes were differentially expressed across the diel cycle of CAM photosynthesis in *A. americana* (Figure 2B). In addition, *GBHM01008063.1* and *GBHM01016483.1* tended to perform opposite expression patterns, compared with *GBHM01026142.1* and *GBHM01043948.1*.

### 3.4. Expression of *SAUR* Genes in Agave during Leaf Development

**Figure 3:**
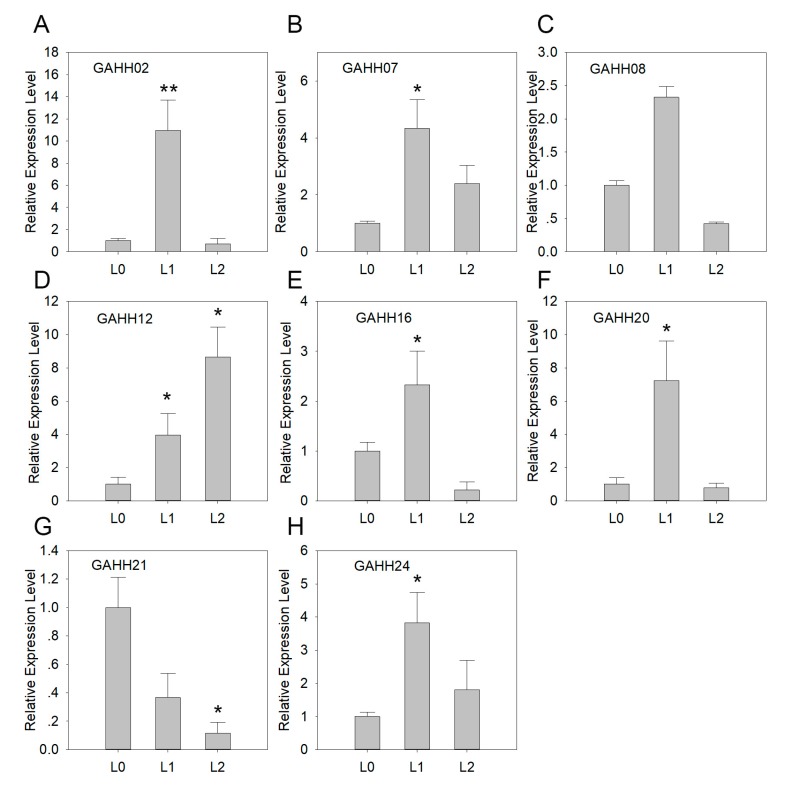
Expression patterns of *GAHH02* (**A**), *GAHH07* (**B**), *GAHH08* (**C**), *GAHH12* (**D**), *GAHH16* (**E**), *GAHH20* (**F**), *GAHH21* (**G**), and *GAHH24* (**H**) at different leaf developmental stages in *A.* H11648 by qRT-PCR. *Y*-axis represents relative expression level. L0, L1 and L2 of *x*-axis represent shoot, unexpanded leaf and expanded leaf, respectively. The error bar represents the standard error. * and ** represent that expression level was increased or decreased by more than 3-fold and 10-fold, respectively (compared with shoot).

*A*. H11648 was selected for further qRT-PCR analysis, and we firstly estimated *SAUR* expression patterns at different leaf developmental stages (Figure 3). Compared with shoot, the expression of six of *SAUR* genes were increased in unexpanded leaf and then decreased in expanded leaf. Among these, four were significantly increased in unexpanded leaves and *GAHH16* was significantly decreased in expanded leaf. Besides, the expression of *GAHH12* was significantly increased during the process, while *GAHH21* was significantly decreased in both unexpanded and expanded leaf. Furthermore, only four genes had significantly decreased expressions in expanded leaf compared with unexpanded leaf.

### 3.5. Expression of Agave *SAUR* Genes under Abiotic and Biotic Stresses

**Figure 4:**
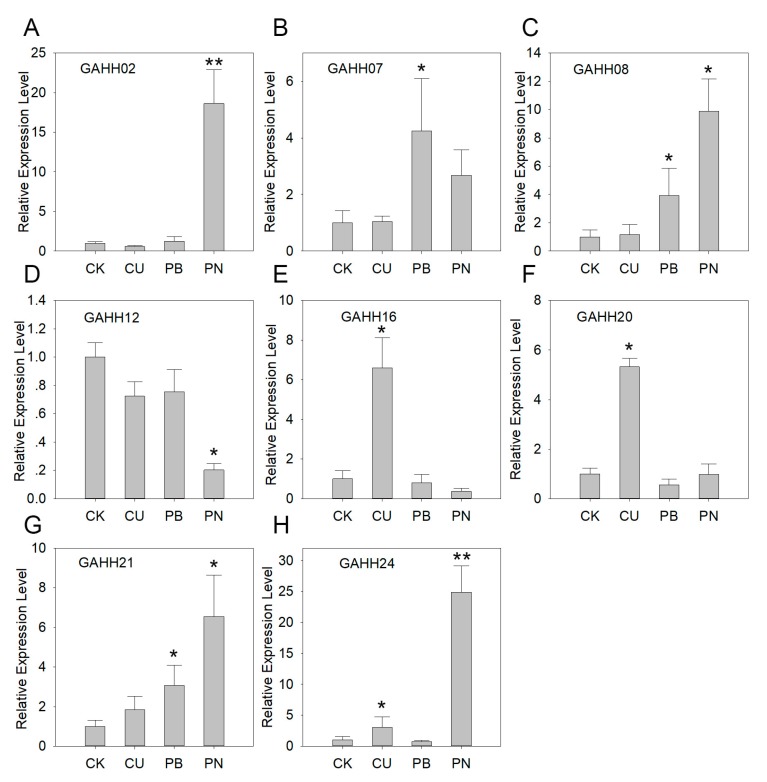
Expression patterns of *GAHH02* (**A**), *GAHH07* (**B**), *GAHH08* (**C**), *GAHH12* (**D**), *GAHH16* (**E**), *GAHH20* (**F**), *GAHH21* (**G**). and *GAHH24* (**H**) under abiotic (Cu and Pb) and biotic (*Phytophthora nicotianae* Breda) stresses in *A.* H11648 by qRT-PCR. *Y*-axis represents relative expression level. CK, CU, PB and PN of *x*-axis represent control, CuSO_4_ treatment, Pb(NO_3_)_2_ treatment and *Phytophthora nicotianae* Breda inoculation, respectively. The error bar represents the standard error. * and ** represent that expression level was higher or lower expressed by more than 3-fold and 10-fold, respectively (compared with control).

*A*. H11648 has a high tolerance to Cu and Pb stresses and *Phytophthora nicotianae* Breda was its main pathogen in cultivation. Thus, the two abiotic stresses and one biotic stress were carried out to evaluate *SAUR* expressions in *A*. H11684 leaves, respectively. Five genes were differentially expressed under one of these stresses, i.e. *GAHH16* and *GAHH20* under Cu stress, *GAHH07* under Pb stress and *GAHH02* and *GAHH12* under biotic stress (Figure 4). The other three genes were highly expressed under the biotic stress and CuSO_4_/Pb(NO_3_)_2_ treatment.

## 4. Discussion

### 4.1. Identification and Evolution of Agave *SAUR* Genes

In this study, we successfully identified 148 *SAUR* genes with full-length coding regions in four *Agave* species, indicating the high efficiency of RNA-Seq for genome mining [31]. Different amounts of *SAUR* genes were obtained in the four *Agave* species. Especially in *A. tequilana*, it had relatively more *SAUR* genes than rice. It was predictable that the large *Agave* genomes could have more *SAUR* genes than rice, which might be caused by the whole genome duplications [5,32]. The phylogenetic analysis depicted a species-specific expansion pattern of *SAUR* gene family in *Arabidopsis* and rice (Figure 1), which was consistent with previous study [22]. In contrast, no similar expansion pattern was observed in the *Agave* species, implying that the transcriptome data might not cover the whole *SAUR* gene family in *Agave* genomes, and the tissue-specific expression of *Agave SAUR* genes as well. Several kinds of tissues were sequenced in *A. tequilana* (4) than in *A. deserti* (3), while in *A*. H11648 and *A. americana*, only leaves were sequenced with the results that were positively correlated with the numbers of *SAUR* gene identified in the four *Agave* species [31,33,34]. However, these results were limited to explain the evolution of *Agave SAUR* genes. Although the availability of *Agave* genome information could partially explain the evolutionary story, it is very difficult to assemble such large *Agave* genomes [32]. The recently published walnut genomes have provided a new clue for the assembly of large and heterozygotic genomes [46]. Taken together, too many challenges limit the understanding of the mechanism of *SAUR* evolution, which needs further investigation.

### 4.2. Candidate *SAUR* Genes Involved in Agave Leaf Development and Stress Response

It has been reported that *SAUR* family is involved in plant growth and developmental processes [29,30], while few *SAUR* genes have been reported in *Agave* species. In fact, *SAUR* genes have crucial roles in plant growth and development throughout the *Agave* lifespan. The in silico expression of *Agave SAUR* tetrads revealed a distinct expression pattern in *A*. H11648 (Figure 2A), irrespective of the *SAUR* gene not being positively selected during *Agave* domestication [36]. Interestingly, *Agave SAUR* genes were differentially expressed across the diel cycle of CAM photosynthesis (Figure 2B), suggesting the existence of a potential relation between auxin signaling and CAM photosynthesis in *Agave*. It is possible that the diel expressions are related to the opening and closings of stomatal cells [34,47], implying that SAUR involved auxin signaling might participate in this process. Moreover, the potential functions of *SAUR* genes in starch accumulation might contribute to the expression pattern [20,47].

We further examined their expression during leaf development of *A*. H11648 and found that all the eight *SAUR* genes were differentially expressed at least at one developmental stage (Figure 3). This finding indicates the *SAUR* genes have potential functions during leaf development. As a kind of leaf fiber crop, leaf development covers the process of fiber development in *A*. H11648. The differentially expressed *SAUR* genes are most likely associated with cell elongation and expansion [6,7], which therefore introduces a new view for further studies on fiber development in *A*. H11648.

As effectors of environmental signals in plant growth and developmental processes, *SAUR* genes are also involved in salt stress responses in rice [48]. And the histidine-rich *AtSAUR30* has a metal-binding capacity, which suggests *SAUR* genes are associated with heavy metal stress as well [49]. Therefore, we performed the Cu and Pb treatments, and found that each stress caused the significant up-regulation of three *SAUR* genes in *A*. H11648 (Figure 4). Surprisingly, none of the six genes were differentially expressed under both stresses and they didn’t contain histidine-rich region. This may be due to the occurrence of different regulations between Cu and Pb stress responses in *A*. H11648. In addition, the main pathogen of *A*. H11648, *Phytophthora nicotianae* Breda was inoculated on leaves to estimate *SAUR* expression patterns. Five genes were differentially expressed during this process implying that these genes might be related to auxin homeostasis–regulated cell wall integrity, and cell wall-mediated immunity [50]. Altogether, three differentially expressed *SAUR* genes under both abiotic and biotic stresses also indicate an interaction between these stresses. It has been reviewed that heavy metal stresses directly affect plant responses by modulating auxin homeostasis [51]. Therefore, these three genes might be involved in heavy metal responses and plant cell wall-mediated immunity. In the future, further functional characterization of these candidate *SAUR* genes could potentially enrich our understanding of their functional diversity.

## 5. Conclusions

In our study, we presented the first identification and expression analysis of *SAUR* genes in *Agave* based on previous transcriptome datasets. About 43, 60, 24, and 21 *SAUR* genes with full-length coding regions were characterized in *A. deserti*, *A. tequilana*, *A*. H11648, and *A. americana*, respectively. The difference observed in tissue-specific transcriptome datasets might be reasoned to the distinct amounts of *SAUR* genes in the four *Agave* species and the tissue-specific expression of *SAUR* genes. Phylogenetic analysis revealed a species-specific expansion pattern of *SAUR* gene family in rice, while no similar phenomenon was observed in *Agave* species. Genome information is still needed to further investigate the duplication and the evolution of *Agave SAUR* genes. The in silico expression shows a distinct expression pattern of *SAUR* genes in *A*. H11648 compared with other *Agave* species. According to the expression analysis, the differentially expressed *SAUR* genes during leaf development might contribute to leaf fiber development of *A*. H11648. Besides, the stress-induced expression patterns of *SAUR* genes demonstrate their potential functions under abiotic and biotic stresses, which also indicates the potential interactions among these stresses. Therefore, further functional characterization of these candidate *SAUR* genes could contribute meaningfully our understanding of their functional diversity.

## Figures and Tables

**Table 1:** Numbers of *Agave Small Auxin Up-regulated RNA* (*SAUR*) genes located at different subcellular positions.

Subcellular Position	*A. deserti*	*A. tequilana*	*A*. H11648	*A. americana*
Chloroplast	3	3	2	1
Cytoplasm	7	3	4	2
Extracellular	0	2	0	0
Mitochondria	12	16	7	7
Nucleus	20	34	9	10
Plasma Membrane	1	2	2	1
Total	43	60	24	21

**Table 2:** Numbers of rice and *Agave SAUR* genes in groups I-VII.

Species	I	II	III	IV	V	VI	VII	VIII	Total
*A. thaliana*	8	5	11	36	3	5	6	5	79
*O. sativa*	5	8	18	3	1	2	16	3	56
*A. deserti*	4	5	3	8	0	1	15	7	43
*A. tequilana*	9	10	8	6	0	5	12	10	60
*A*. H11648	3	2	4	5	0	1	5	4	24
*A. americana*	2	1	5	3	0	2	4	4	21

## Supplementary Materials

The following are available online at https://www.mdpi.com/2073-4425/10/7/555/s1, Figure S1: Alignment of *SAUR* proteins in the five plant species, Table S1: Primers for qRT-PCR analysis. Table S2: Details of *SAUR* genes in *Agave* species.
