# Supplementary figures and images for: Identification and Expression of SAUR Genes in the CAM Plant Agave
Source: Genes (Basel). 2019 Jul 23;10(7):555. doi: 10.3390/genes10070555 (PMC6679190; doi:10.3390/genes10070555)

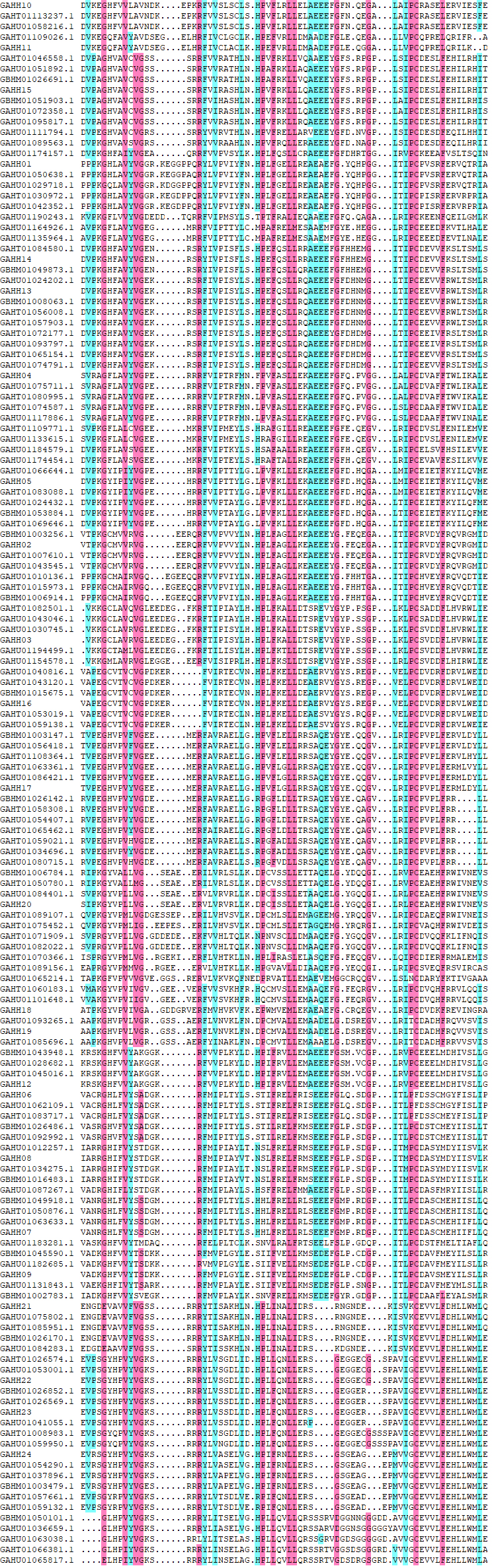

Supplement: Supplementary file 1 [file genes-10-00555-s001.zip › Figure S1.jpg]
